# Prefrontal Cortex Dysregulation of Amino Acid–Glucose Homeostasis Links High-Fat and/or High-Fructose Intake to Cognitive Deficits in Male Mice

**DOI:** 10.1007/s11064-026-04791-x

**Published:** 2026-06-02

**Authors:** Humberto Martínez-Orozco, Luis Antonio Reyes-Castro, Consuelo Lomas-Soria, Silvia Solís-Ortíz, Sofía Yolanda Diaz-Miranda

**Affiliations:** 1 Laboratorio de Neuromorfometría y Desarrollo, Instituto de Neurobiología, Universidad Nacional Autónoma de México, Campus UNAM, Boulevard Juriquilla 3001, Querétaro, México; 2 Departamento de Biología de la Reproducción, Instituto Nacional de Ciencias Médicas y Nutrición Salvador Zubirán, Vasco de Quiroga 15, Ciudad de México, México; 3 Departamento de Ciencias Médicas, División de Ciencias de la Salud, Universidad de Guanajuato, Campus León, León, Guanajuato México

**Keywords:** Prefrontal cortex, Metabolism, Neurotransmitter regulation, Glucose metabolic pathways, Obesity, Memory dysfunction

## Abstract

Memory processes are susceptible to impairment induced by excessive consumption of hypercaloric diets, particularly those rich in saturated fats and fructose. Such dietary patterns have been linked to disrupted neurotransmission in the prefrontal cortex (PFC), where the balance between excitation and inhibition depends on efficient glucose metabolism and the synthesis of neuroactive amino acids. However, the molecular mechanisms underlying these effects remain poorly understood. Here, male C57BL/6 mice were fed for 10 weeks with a control diet, a high-fat diet (HFD), a high-fructose diet (HFrD), or a combined high-fat/high-fructose diet (HFFrD). Their body weight gain and visceral adiposity were primarily driven by saturated fat intake, whereas hyperglycemia was observed across all diets. Also, we assessed metabolic outcomes, recognition memory, and PFC molecular profiles, including neuroactive amino acids (GABA, glutamate, glutamine, aspartate, alanine, glycine, and taurine) and the expression of genes related to glucose metabolism (*Slc2a1*, *Pcx*, *G6pd*, *Gck*, *Pck1*, *Irs2*) and the glutamate/GABA–glutamine cycle (*Glul*, *Glud1*, *Gad1*, *Gad2*). Behaviorally, HFFrD reduced locomotor activity and caused the most significant impairment in recognition memory. In the PFC, diet composition generated distinct amino acid profiles, revealing vulnerability of the glutamine–glutamate–GABA cycle to hypercaloric intake. Transcriptional responses were diet-specific, with consistent *Gad1* upregulation and broader induction of glucose metabolism–related genes. Together, these findings demonstrate diet-dependent metabolic and neurochemical remodeling in the PFC and support a link between excessive fat and/or fructose intake with disrupted glutamine–glutamate homeostasis and memory deficits.

## Introduction

Obesity affects nearly half of the adult population worldwide and, alarmingly, also impacts billions of adolescents and children, positioning this condition as one of the most urgent global public health concerns [1]. A major contributor to the rising prevalence of obesity is overnutrition, driven by the increased availability and accessibility of hypercaloric foods within households; consumer preferences for sweet, fatty/fried foods and salty snacks; irregular eating habits; sedentary behavior; and chronic exposure to stressful environments [2]. In particular, dietary patterns characterized by high intake of sugar-sweetened beverages, fast food, bakery products, confectionery, and processed salty snacks have been strongly linked to overweight and obesity in diverse populations [3, 4]. These products, often referred to as palatable foods, are typically rich in saturated fats and added sugars—especially fructose—forming dietary regimens commonly classified as high-fat diets (HFDs), high-fructose diets (HFrDs), or high-fat–high-fructose diets (HFFrDs).

Excessive intake of saturated fats and fructose promotes body fat accumulation and induces profound metabolic and physiological alterations, leading to dysfunction at the peripheral level, like adipose tissue dysregulation, activation of inflammatory pathways, altered expression of catabolic-related genes, impaired insulin signaling and glucose homeostasis, disruptions in lipid metabolism, and leptin resistance [5–7]. Eventually, hypercaloric diets rich in these nutrients induce central alterations, such as reductions in the volume of specific brain regions, as well as neuroinflammation and detrimental effects on synaptic function, all of which negatively impact vital brain processes [8–11].

Among the brain regions affected by diet-induced obesity, the prefrontal cortex (PFC) has received particular attention due to its central role in regulating the intake of highly palatable foods [12] and reward-driven overeating behaviors that contribute to obesity development [13]. PFC neural activity is essential for self-regulatory control and decision-making processes [14], as well as for emotional processing, including affective responses to food-related cues and risk evaluation [15, 16]. In addition, the PFC is implicated in higher-order cognitive functions such as episodic memory formation [17] and spatial learning [18]. Disruption of these functions may lead to premature cognitive decline and increase vulnerability to neurodegenerative disorders associated with chronic consumption of palatable, hypercaloric diets [19, 20].

The mechanisms through which diet-induced alterations in the PFC contribute to impairments in brain functions, such as memory, remain incompletely understood. Evidence from rodent studies indicates that HFDs alter the expression of genes encoding proteins involved in neurogenesis, synaptic function, and calcium signaling pathways [21], suggesting that transcriptional remodeling may underlie the detrimental effects of these diets on neural plasticity [22] and subsequently memory decline [10, 19]. Notably, glucose and amino acid homeostasis are two key physiological processes that strongly influence PFC-dependent memory formation. Neuroimaging studies have shown that brain glucose concentrations are positively associated with peripheral glucose levels in obesity and diabetes [23]. In parallel, adequate cerebral glucose availability is essential for neurons and astrocytes to sustain optimal levels of neuroactive amino acids through the glutamine–glutamate–gamma-aminobutyric acid (GABA) cycle [24]. Diets rich in fats and fructose have been reported to modify the expression of glucose transporters such as GLUT1, encoded by the *Slc2a1* gene, reflecting altered cerebral glucose uptake [25–29]. These metabolic alterations may disrupt cortical concentrations of key neurotransmitters, including glutamate and GABA, as well as other neuroactive amino acids such as glutamine, alanine, and aspartate [30–33].

Substantial advances have been made in understanding the metabolic fate of glucose within the glutamine–glutamate–GABA cycle [24], which is useful for developing research focused on brain metabolism in health and disease. Physiologically, glucose uptake in the brain is primarily mediated by astrocytes via GLUT1. Once internalized, glucose undergoes glycolysis to generate pyruvate, which can be carboxylated by pyruvate carboxylase (PC; encoded by the *Pcx* gene) to form oxaloacetate [34]. Oxaloacetate serves as a key anaplerotic intermediate of the tricarboxylic acid (TCA) cycle and is essential for sustaining both the glutamine–glutamate and glutamine–GABA cycles [24]. Within the TCA cycle, α-ketoglutarate derived from PC-dependent anaplerosis is converted into glutamate and subsequently glutamine through the coordinated actions of glutamate dehydrogenase (encoded by *Glud1*) and glutamine synthetase (encoded by *Glul*) [35, 36]. GABAergic neurons take up astrocyte-derived glutamine, which is then converted to GABA by glutamic acid decarboxylase (GAD). This enzyme exists as two isoforms: GAD65, predominantly localized in the cytoplasm and encoded by *Gad1*, and GAD67, primarily associated with synaptic terminals and encoded by *Gad2* [37]. Additionally, depending on cellular metabolic demands, astrocytic glucose may be diverted toward gluconeogenesis or the pentose phosphate pathway (PPP) [38–40, 41]. These alternative metabolic routes have important implications for the synthesis and regulation of neuroactive amino acids such as aspartate, alanine, glycine, and taurine, which can further modulate the glutamine–glutamate–GABA cycle and neuronal excitability [42–47].

Given that glutamate and GABA levels are essential for PFC-mediated functions such as memory [48–51], it is highly relevant to characterize the impact of hypercaloric diets on proteins and amino acids involved in excitatory and inhibitory neurotransmission. This is important because studies addressing these mechanisms, particularly those involving glucose metabolism in the PFC, remain limited. Accordingly, this study aimed to evaluate the effects of consuming saturated fats, fructose, and their combination on the transcriptional remodeling of proteins associated with glucose–amino acid homeostasis, as well as on the concentrations of neuroactive amino acids in the PFC of mice. Furthermore, this work sought to determine whether these molecular alterations are associated with memory deficits.

## Materials and Methods

### Experimental Animals

A total of sixty healthy 13-week-old male C57BL/6 mice from different litters were obtained from the vivarium of the Institute of Neurobiology, UNAM (Mexico), and used in this study. Animals were randomly housed in polypropylene cages (2–3 mice per cage) under controlled environmental conditions (22 ± 2 °C) and maintained on a 12 h light/12 h dark cycle. Prior to dietary intervention, mice were allowed a one-week acclimation period with ad libitum access to standard laboratory chow (Purina^®^ 5001) and water. Male mice were selected to reduce biological variability associated with hormonal fluctuations across the estrous cycle in females, which can influence diet-induced obesity [52] but also neurochemical effects like GABA and glutamate synthesis modulated by estrogens [53]. All experimental procedures were conducted in the facilities of the University of Guanajuato Campus Leon in accordance with the National Research Council’s Guide for the Care and Use of Laboratory Animals and were approved by the Institutional Bioethics Committee of the University of Guanajuato (Approval Number CIBIUG-P36-2018).

### Dietary Intervention

All experimental diets were custom-formulated and produced by the National Institute of Medical Sciences and Nutrition Salvador Zubirán (Mexico City, Mexico), following previously validated protocols [54]. Diet composition was defined based on the percentage of total caloric content derived from macronutrients. The control diet (CD) provided 11.0% of total calories from fat, 69.0% from carbohydrates, and 20.0% from protein. The HFD consisted of 48.0% of calories from fat, 33.0% from carbohydrates, and 19.0% from protein. The HFrD contained 11.0% of calories from fat, 69.0% from carbohydrates—of which 33.0% of total calories were specifically from fructose—and 20.0% from protein. The HFFrD provided 48.0% of calories from fat, 33.0% from carbohydrates (exclusively in the form of fructose), and 19.0% from protein. Detailed diet composition, including caloric density and ingredient formulation, is provided in Table 1.

**Table 1:** Composition of experimental diets

	CD	HFD	HFrD	HFFrD
Casein	190	226	190	226
L-cystine	3	3	3	3
Corn starch	502	296	172	0.0
Maltodextrin	146	113	146	0.0
Fructose	0	0	330	409
Cellulose	50	50	50	50
Soy oil	28	50	28	50
Lard	19	200	19	200
Mineral mix	50	50	50	50
Vitamin mix	10	10	10	10
Choline bitartrate	2	2	2	2

The content of each nutrient is expressed in grams per kilogram. *CD* control diet, *HFD* high-fat diet, *HFrD* high-fructose diet, *HFFrD* high-fat high/fructose diet

At 14 weeks of age, mice from every set were randomly allocated to one of four experimental groups (CD, *n* = 14; HFD, *n* = 14; HFrD, *n* = 16; or HFFrD, *n* = 16) and maintained on their respective diets for 10 weeks. Animals had unrestricted access to food and water (ad libitum) throughout the experimental period. Body weight and food intake were measured weekly. Any animal that exhibited sudden and critical weight loss during the dietary intervention was excluded from the experiment. At the end of the dietary intervention, mice were euthanized in accordance with institutional ethical guidelines, and tissues were rapidly collected and processed following the protocols described below.

### Tissue Processing

Mice were fasted for 8 h prior to the experimental procedures and subsequently anesthetized with pentobarbital (60 mg/kg, i.p.). Blood glucose levels were measured in deeply anesthetized animals using Accu-Chek^®^ Instant test strips and a portable glucometer after collecting a small blood sample from the tip of the tail. Immediately thereafter, animals were rapidly dissected to excise and weigh epididymal adipose tissue. The brain was quickly removed, and the PFC was dissected (approximately, AP + 3.20 to + 2.22 relative to Bregma [55]) on an ice-cold glass surface based on a previous protocol [56], weighed, and stored in cryogenic tubes at − 80 °C until further analysis. The entire procedure was completed within 3–5 min to minimize bias related to neurochemical changes derived from post-mortem ischemia [57].

### Quantification of Neuroactive Amino Acids

Concentrations of GABA (Standard, Catalog # A2129, Sigma-Aldrich), glutamate (Standard, Catalog # G1251, Sigma-Aldrich), glutamine (Standard, Catalog #G3126, Sigma-Aldrich), aspartate (Standard, Catalog # A9256, Sigma-Aldrich), alanine (Standard, Catalog # A7627, Sigma-Aldrich), glycine (Standard, Catalog # G7126, Sigma-Aldrich), and taurine (Standard, Catalog # 86329, Sigma-Aldrich) were quantified by high-performance liquid chromatography (HPLC) coupled with electrochemical detection using a BASS LC-4 C system equipped with an amperometric detector, as previously described [54]. Briefly, PFC samples (*n* = 6 per group) were slowly thawed on ice and homogenized in an ice-cold solution containing methanol (Catalog #34860, Sigma-Aldrich) and 0.1 M phosphate-buffered saline (pH 7.4, 137 mM NaCl) at a 1:1 ratio. Tissue homogenization was performed using ultrasonication to obtain a final concentration of 200 mg of tissue per milliliter. Homogenates were then centrifuged at 13,500 rpm for 20 min at 4 °C, and the resulting supernatants were collected and filtered. Amino acids were derivatized with O-phthaldialdehyde (Catalog #79760, Sigma-Aldrich) and subsequently separated by HPLC with electrochemical detection. Individual amino acid concentrations were calculated using external standards and normalized to micromoles per gram of wet tissue.

### Transcriptional Quantification

PFC samples were collected immediately after euthanasia for the analysis of gene expression related to the regulation of the glutamate/GABA–glutamine cycle. Target genes included *Glul* (glutamine synthetase), *Gad1* (glutamate decarboxylase 65), *Gad2* (glutamate decarboxylase 67), and *Glud1* (glutamate dehydrogenase 1). In parallel, the expression of genes involved in glucose metabolism and cellular energy regulation was assessed, including *Pcx* (pyruvate carboxylase), *Slc2a1* (glucose transporter 1), *G6pd* (glucose-6-phosphate dehydrogenase), *Gck* (glucokinase), *Pck1* (phosphoenolpyruvate carboxykinase 1), and *Irs2* (insulin receptor substrate 2).

mRNA levels were quantified as previously described [54]. Briefly, total RNA was isolated from PFC tissue using TRIzol™ reagent (Catalog #15596026, Invitrogen), with five biological replicates per group (*n* = 5 per group). RNA samples were reverse transcribed using a commercial reverse transcription kit (Catalog #K1642, Thermo Scientific). Quantitative PCR (qPCR) was performed under identical conditions for all target genes and normalized to the housekeeping gene *Rpl32*, used as an internal control. Primer sequences are listed in Table 2. Amplification was carried out using a LightCycler^®^ 2.0 real-time PCR system (Roche) with Roche master mix (Catalog #04707494001, Roche) and hydrolysis probes (Universal Probe Library; Catalog #04683633001, Roche). Relative gene expression levels were calculated using the ΔΔCT method.

**Table 2:** Primers and probes used in reverse transcription real-time quantitative polymerase chain

Gene	Forward sequence
*Glul*	F: 5′-TTCAAGTGGGAACTTGCTGA-3′ R: 5′-CTGGCTCTCCTGACCTGTTC-3′
*Gad1*	F: 5′-CTTGGCGTAGAGGTAATCAGC-3′ R: 5′-CAAGTTCTGGCTGATGTGGA-3′
*Gad2*	F: 5′-GCATGGCATACATGTTGGAG-3′ R: 5′-ACTAAAGAAAATGAGAGAAATCATTGG-3′
*Glud1*	F: 5′-GCTTCTGCTCCTCGCTCT-3′ R: 5′-GCCAGCATCGTAGAGGAC-3′
*Pcx*	F: 5′-CAGGAACTGCTGGTTGTTGA-3′ R: 5′-TCCGTGTCCGAGGTGTAAA-3′
*Slc2a1*	F: 5′-TCCCACAGCCAACATGAG-3′ R: 5′-TTACAGCGCGTCCGTTCT-3′
*G6pd*	F: 5′-ATCTTCACACCACTGCTGCA-3′ R: 5′-CCGCGGCTGCCATATACATA-3′
*Gck*	F: 5′-ATCCGGGAAGAGAAGCAAGC-3′ R: 5′-GATGAGGGACAGAGGGACCT-3′
*Pck*	F: 5′-TGCGGATCATGACTCGGATG-3′ R: 5′-GGCACTTGATGAACTCCCCA-3′
*Irs2*	F: 5′-CGCCACAGTTCAGAGACCTT-3′ R: 5′-CGCTTGGAATTGTGGGCAAA-3′
*Rrpl32*	F: 5′-GCTGCCATCTGTTTTACGG-3′ R: 5′-TGACTGGTGCCTGATGAACT-3′

### Cognitive Assessment

Memory performance was assessed in a set of mice (*n* = 7–8 per group) for all experimental groups using the novel object recognition task, following a previously established protocol [54]. This test evaluates recognition memory of rodents, a type of memory involved in the identification of previously encountered stimuli (for instance, objects) leveraging their natural preference or novelty [58], which requires participation of PFC [59, 60]. Briefly, the procedure consisted of three phases separated by 24 h intervals: habituation, familiarization, and test. During the habituation phase, mice were allowed to explore the empty apparatus (open field) freely. In the familiarization phase, animals were exposed to two identical objects. In the test phase, one of the familiar objects was replaced with a novel object. The apparatus consisted of an arena (40 × 40 × 40 cm). Behavioral activity was video-recorded via a computer-based interface. Video files were analyzed manually using a chronometer to quantify object exploration times, which were subsequently used to calculate the percentage of time spent exploring each object and the discrimination index, as previously described [61]. The discrimination index was calculated with the formula: (time in novel - time in familiar)/(total exploration time).

### Statistical Analysis

Statistical analyses were performed using GraphPad Prism version 8.0.3 for Windows (GraphPad Software, Inc.). Prior to inferential testing, data were assessed for normality using the Kolmogorov–Smirnov test. A repeated-measures one-way analysis of variance (ANOVA) was applied to body weight data. For the other dependent variables, a one-way ANOVA was used to evaluate the effects of diet. When appropriate, post hoc comparisons were performed using Tukey’s or Dunnett’s multiple-comparison tests. Statistical significance was set at *p* < 0.05.

## Results

### Dietary Fat and Fructose Differentially Modulate Body Weight and Metabolic Outcomes in Mice

**Fig. 1:**
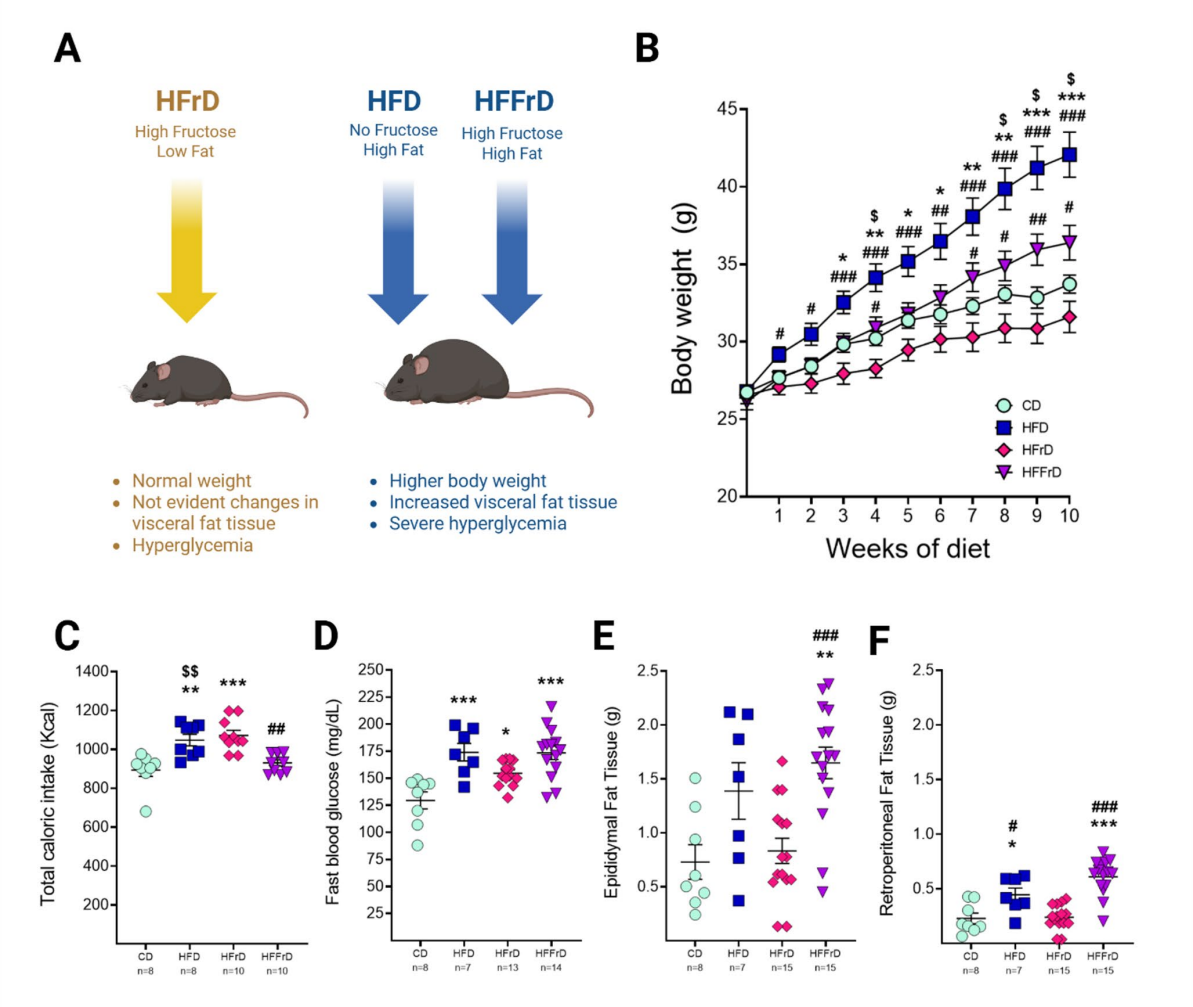
Effects of dietary fat and fructose content on obesity-related outcomes in mice. **A** Summary of dietary intervention characteristics. **B** Body weight trajectories over the 10-week intervention for each dietary group. Grouped bar graphs show total caloric intake **C**, fasting blood glucose levels **D**, and weights of epididymal **E** and retroperitoneal **F** adipose tissues measured at the end of the intervention (week 10). Data are presented as mean ± SEM. Symbols indicate significant post hoc multiple-comparison differences: *p < 0.05, **p < 0.01, ***p < 0.001 vs. CD; #*p* < 0.05, ##*p* < 0.01, ###*p* < 0.001 vs. HFrD; $*p* < 0.05, $$*p* < 0.01 vs. HFFrD. *CD* control diet, *HFD* high-fat diet, *HFrD* high-fructose diet, *HFFrD* high-fat/high-fructose diet

The main findings of the effects of dietary fat and fructose content on obesity-related outcomes in our mice are shown in Fig. 1. Repeated-measures ANOVA revealed significant main effects of the diet group [F(3,56) = 12.72, *p* < 0.001] and treatment duration [F(1.926,107.8) = 238.6, *p* < 0.001], as well as a significant group × time interaction [F(30,560) = 12.88, *p* < 0.001], indicating that body weight trajectories differed depending on the dietary condition. Sidak’s multiple comparisons test showed that mice fed the HFD exhibited significantly greater body weights compared with control animals from week five through the end of the intervention (Fig. 1B). No significant differences were observed between the HFrD or HFFrD groups and controls at any time point, suggesting that fructose intake attenuated both fat-induced and baseline body weight gain in male mice. Consistent with this observation, body weight in HFrD-fed animals was significantly lower than in HFFrD-fed mice from week seven onward and lower than in HFD-fed mice from week two until the end of the treatment.

One-way ANOVA revealed a significant effect of diet on total caloric intake [F(3,32) = 11.44, *p* < 0.001]. Post hoc analyses showed that mice fed HFD (*p* = 0.001) and HFrD (*p* < 0.001) consumed more calories than CD-fed animals. In contrast, caloric intake in the HFFrD group was lower than in both HFD (*p* = 0.009) and HFrD (*p* = 0.001) groups (Fig. 1C).

With respect to metabolic parameters, diet significantly influenced fasting blood glucose [F(3,38) = 9.981, *p* < 0.001], epididymal adipose tissue mass [F(3,41) = 8.247, *p* < 0.001], and retroperitoneal adipose tissue mass [F(3,41) = 21.21, *p* < 0.001]. Mice in the HFrD group did not exhibit significant increases in epididymal or retroperitoneal fat compared with CD-fed mice, consistent with their stable body weight (Fig. 1E, F). However, fasting blood glucose levels were elevated (Fig. 1D), indicating that HFrD-induced hyperglycemia may occur independently of visceral fat accumulation. In contrast, the HFD increased retroperitoneal fat relative to the CD (*p* = 0.026) and HFrD (*p* = 0.015). The HFFrD had a more pronounced effect, with higher epididymal (*p* = 0.002 vs. CD; *p* < 0.001 vs. HFrD) and retroperitoneal fat mass (*p* < 0.001 vs. CD; *p* < 0.001 vs. HFrD) (Fig. 1E, F). Additionally, both the HFD and HFFrD significantly increased fasting blood glucose compared with the CD (*p* < 0.001) (Fig. 1D).

Overall, these findings underscore a predominant role of excessive saturated fat intake in promoting visceral adiposity and hyperglycemia, effects that appear attenuated when fructose is consumed alone.

### Diet Composition Differentially Alters Neuroactive Amino Acid Homeostasis in the Prefrontal Cortex

**Fig. 2:**
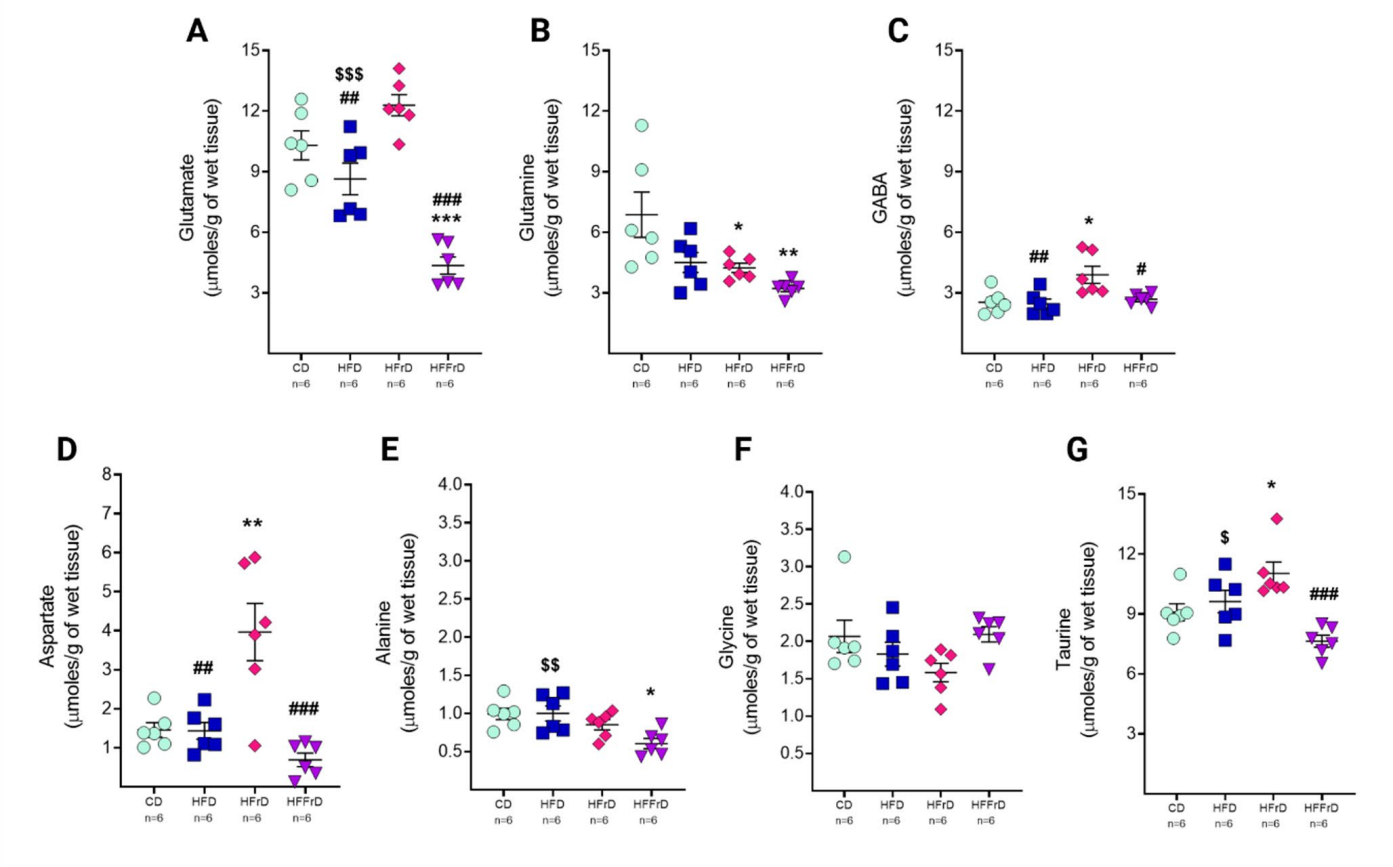
Effects of dietary fat and fructose content on neuroactive amino acids in the prefrontal cortex. Micromolar concentrations of **A** glutamate, **B** glutamine, **C** GABA, **D** aspartate, **E** alanine, **F** glycine, and **G** taurine are shown in grouped dot plots. All data were normalized per gram of wet tissue and are presented as mean ± SEM. Symbols indicate significant post hoc multiple-comparison differences: *p < 0.05, **p < 0.01, ***p < 0.01 vs. CD; #*p* < 0.05, ##*p* < 0.01, ###*p* < 0.001 vs. HFrD; $*p* < 0.05, $$*p* < 0.01, $$$*p* < 0.001 vs. HFFrD. *CD* control diet, *HFD* high-fat diet, *HFrD* high-fructose diet, *HFFrD* high-fat/high-fructose diet

Micromolar concentrations of neuroactive amino acids measured in PFC homogenates by high-performance liquid chromatography are presented in Fig. 2. One-way ANOVA revealed significant effects of diet on glutamate [F(3,20) = 28.67, *p* < 0.001], glutamine [F(3,20) = 6.050, *p* = 0.004], GABA [F(3,20) = 6.051, *p* = 0.004], aspartate [F(3,20) = 12.59, *p* < 0.001], alanine [F(3,20) = 5.662, *p* = 0.006], and taurine [F(3,20) = 8.742, *p* < 0.001]. Glycine levels were not significantly altered [F(3,20) = 2.314, *p* = 0.107].

The HFD did not produce significant changes in any neuroactive amino acid within the PFC, although trends toward reduced glutamate (*p* = 0.077) and glutamine (*p* = 0.066) were observed (Fig. 2A, B), relative to the CD. However, compared with HFrD-fed mice, HFD animals exhibited lower glutamate (*p* = 0.002), GABA (*p* = 0.007), and aspartate (*p* = 0.001) concentrations (Fig. 2A, C, D), while showing higher levels of glutamate (*p* < 0.001), alanine (*p* = 0.009), and taurine (*p* = 0.036) relative to HFFrD-fed mice (Fig. 2A, E, G).

In contrast, HFrD intake resulted in decreased glutamine (*p* = 0.036) and increased GABA (*p* = 0.011), aspartate (*p* = 0.001), and taurine (*p* = 0.040) compared with CD intake (Fig. 2B–D, G). Glutamate levels also showed a trend toward elevation in the HFrD group relative to controls (*p* = 0.074) (Fig. 2A).

Notably, the HFFrD produced marked reductions in glutamate (*p* < 0.001) and glutamine (*p* = 0.003), along with decreased alanine (*p* = 0.010), compared with the CD (Fig. 2A, B, E).

Overall, these findings demonstrate that diets enriched in fat, fructose, or both differentially alter neuroactive amino acid profiles in the PFC, with pronounced effects on those associated with the glutamine–glutamate cycle.

### High Fat and Fructose Content in the Diet Influences the Regulation of Glucose Metabolism Genes and Consistently Upregulates *Gad1* in the Prefrontal Cortex

**Fig. 3:**
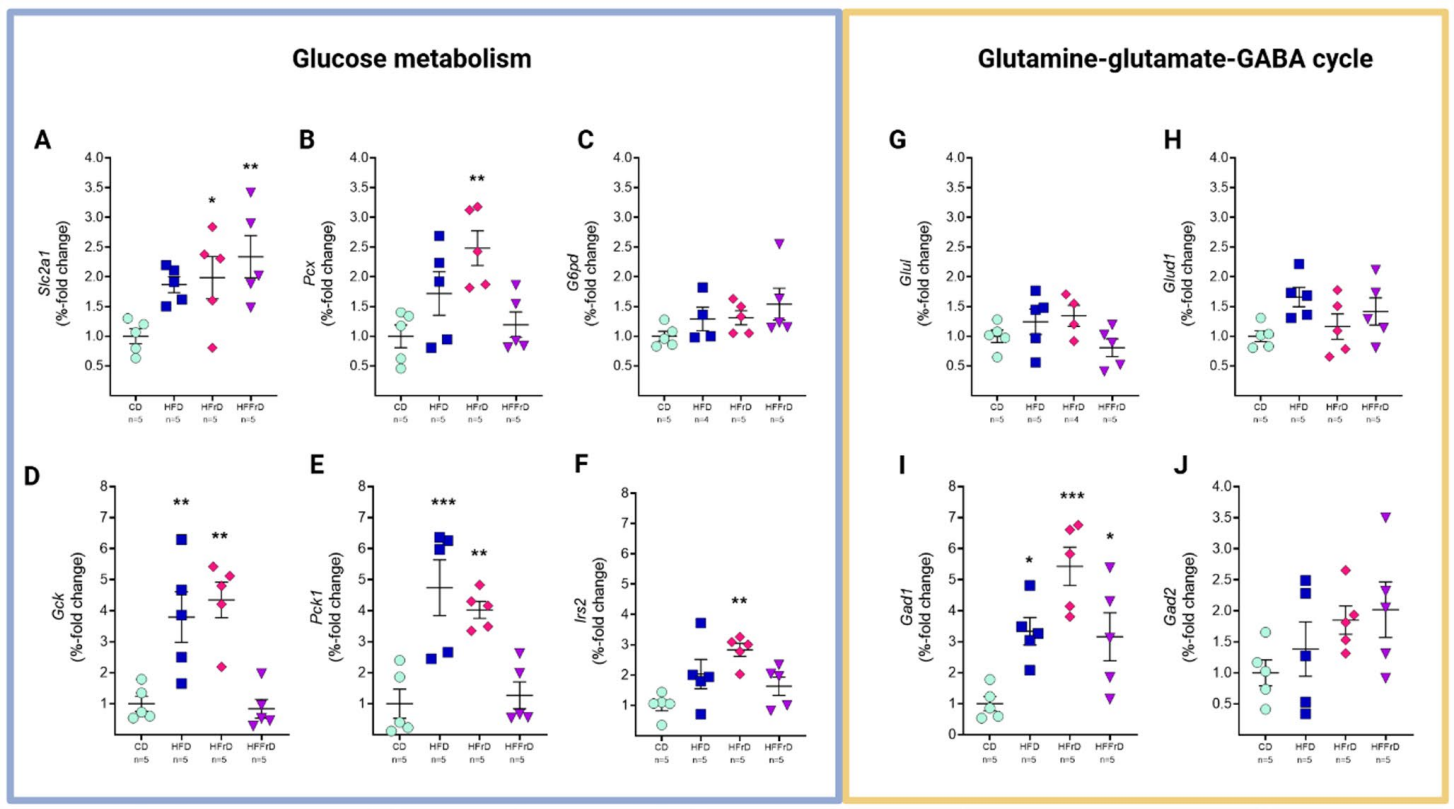
Effects of dietary fat and fructose content on the expression of genes related to glucose and amino-acid homeostasis (glutamate–glutamine–GABA cycle) of the prefrontal cortex. The percentage fold of change in **A** *Slc2a1*, **B** *Pcx*, **C** *G6pd*, **D** *Gck*, **E** *Pck1*, **F** *Irs2*, **G** *Glul*, **H** *Glud1*, **I** *Gad1*, and **J** *Gad2* with respect to the control is shown in grouped dot plots. Quantitative PCR (qPCR) was performed under identical conditions for all target genes and normalized to the housekeeping gene *Rpl32*, which was used as an internal control. All data are presented as mean ± SEM. Symbols indicate significant differences compared to the CD (*p < 0.05, **p < 0.01, ***p < 0.001). *CD* control diet, *HFD* high-fat diet, *HFrD* high-fructose diet, *HFFrD* high-fat/high-fructose diet

Relative fold changes in mRNA expression of genes associated with glucose metabolism and the glutamine–glutamate–GABA cycle in the PFC are shown in Fig. 3. One-way ANOVA revealed significant effects of diet on transcript levels of *Pcx* [F(3,16) = 5.810, *p* = 0.007], *Slc2a1* [F(3,16) = 4.524, *p* = 0.018], *Gad1* [F(3,16) = 10.67, *p* < 0.001], *Gck* [F(3,16) = 11.74, *p* < 0.001], *Pck1* [F(3,16) = 11.24, *p* < 0.001], and *Irs2* [F(3,16) = 5.862, *p* = 0.007]. Dunnett’s post hoc comparisons versus CD revealed diet-specific transcriptional responses, predominantly characterized by upregulation.

In HFD-fed mice, mRNA levels of *Gck* (*p* = 0.005), *Pck1* (*p* < 0.001), and *Gad1* (*p* = 0.023) were significantly increased (Fig. 3D, E, I), with trends toward elevated *Slc2a1* (*p* = 0.088) and *Irs2* (*p* = 0.086) expression (Fig. 3A, F). HFrD induced broader transcriptional changes, increasing *Slc2a1* (*p* = 0.048), *Pcx* (*p* = 0.004), *Gck* (*p* = 0.001), *Pck1* (*p* = 0.005), *Irs2* (*p* = 0.002), and *Gad1* (*p* < 0.001) (Fig. 3A, B, D–F, I). In contrast, HFFrD selectively elevated *Slc2a1* (*p* = 0.007) and *Gad1* (*p* = 0.036).

Collectively, these findings indicate that diets enriched in fat, fructose, or both differentially modulate the transcriptional profile of genes involved in glucose metabolism, while consistently increasing *Gad1* expression in the PFC.

### The Combined Diet Reduces Locomotor Activity and Impairs Recognition Memory Performance

**Fig. 4:**
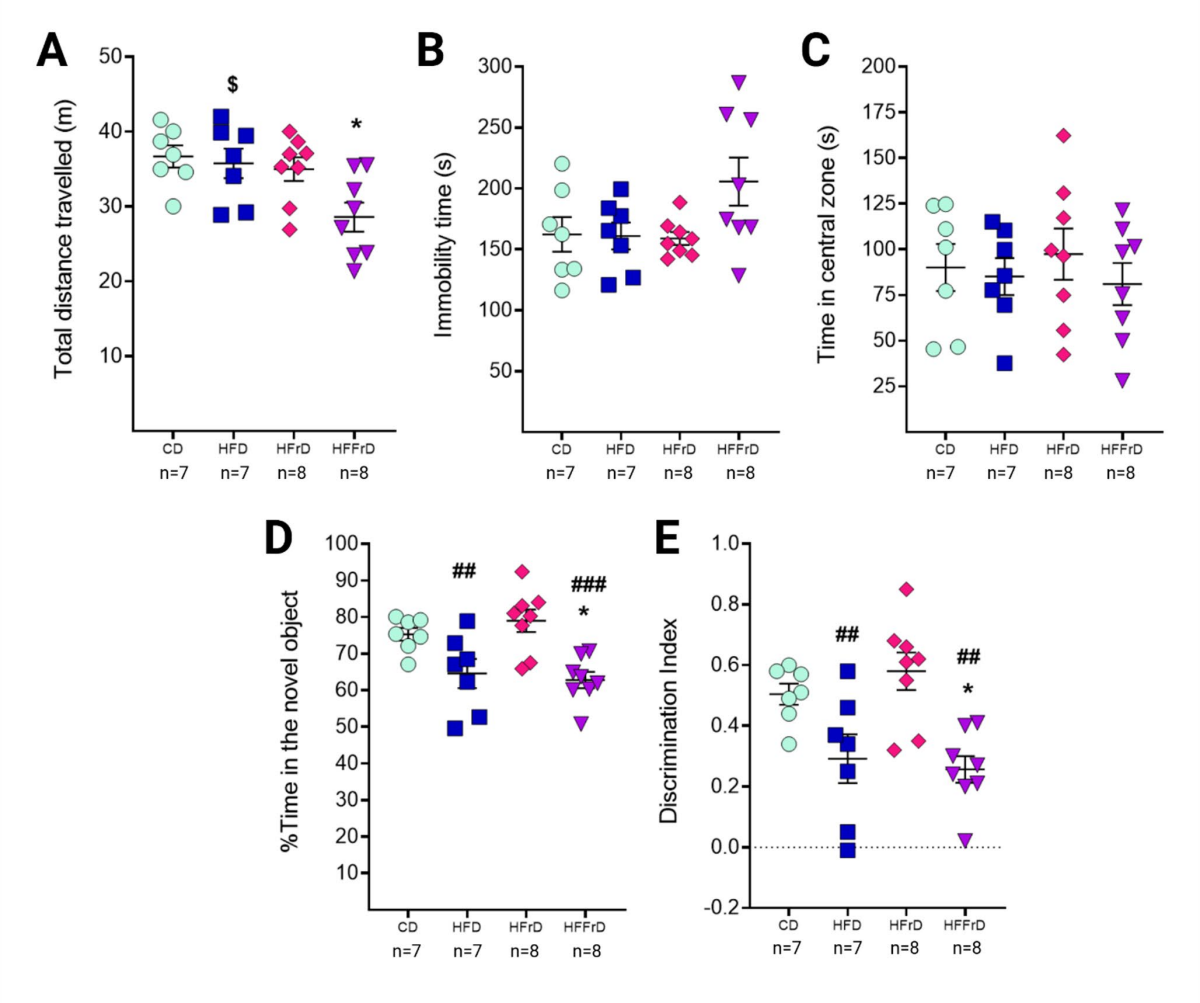
Effects of dietary fat and fructose content on behavioral performance in mice. Locomotor activity and anxiety-related parameters assessed during the habituation phase of the novel object recognition task: **A** total distance traveled in the arena, **B** immobility time, and **C** time spent in the central zone. Recognition memory performance evaluated during the test phase is shown as **D** percentage of time spent with the novel object and **E** discrimination index. Data are presented as mean ± SEM. Statistical analyses were performed using one-way ANOVA. Symbols indicate significant differences between groups: *p < 0.05 vs. CD; ##*p* < 0.01, ###*p* < 0.001 vs. HFrD; $*p* < 0.05 vs. HFFrD. *CD* control diet, *HFD* high-fat diet, *HFrD* high-fructose diet, *HFFrD* high-fat/high-fructose diet

Locomotor activity and recognition memory were evaluated to assess the behavioral impact of the hypercaloric diets. A one-way ANOVA revealed a significant effect of diet on total distance traveled [F(3,26) = 4.501, *p* = 0.011] during the habituation phase of the novel object recognition task (open-field session). Post hoc analysis showed that HFFrD-fed mice traveled significantly shorter distances than mice in the CD (*p* = 0.016) and HFD (*p* = 0.038) groups, with a trend toward reduction compared with HFrD-fed mice (*p* = 0.060) (Fig. 4A). No significant dietary effects were detected for immobility time [F(3,26) = 2.781, *p* = 0.061] or time spent in the central zone [F(3,26) = 0.3450, *p* = 0.793] (Fig. 4B, C). These findings indicate that anxiety-related parameters were not markedly affected by dietary treatments, although HFFrD intake appeared to negatively influence locomotor activity.

Recognition memory performance was also significantly modulated by diet, as reflected by changes in both the percentage of time spent exploring the novel object [F(3,26) = 7.891, *p* < 0.001] and the discrimination index [F(3,26) = 7.834, *p* < 0.001]. HFD-fed mice exhibited trends toward reduced novel object exploration (*p* = 0.074) and discrimination index (*p* = 0.078) relative to CD animals. In contrast, HFFrD-fed mice showed significant reductions in both measures compared with controls (novel object exploration: *p* = 0.024; discrimination index: *p* = 0.025) (Fig. 4D, E). Notably, the HFrD group displayed higher values for both parameters than the HFD and HFFrD groups (*p* < 0.01). Collectively, these findings suggest that high saturated fat intake contributes more substantially than fructose alone to recognition memory impairment, with HFFrD causing more pronounced deficits in long-term memory performance.

## Discussion

Dietary patterns characterized by excessive consumption of fat- and fructose-rich foods contribute to obesity by promoting adipose tissue expansion and dysfunction, leading to increased body weight and metabolic disturbances such as hyperglycemia. In the present study, these alterations were primarily driven, over a relatively short time frame, by high intake of saturated fats. This observation is consistent with previous reports indicating that dietary fat is a major determinant of body weight gain in rodents [62]. In contrast, HFrD induced hyperglycemia without significant changes in body weight or adiposity. Similarly, Togo et al. [63] reported increased adiposity in rodents exposed to sucrose-sweetened water, but not in animals fed an isocaloric solid diet, and identified a positive correlation between glucose intolerance and body fat mass. Together, these contrasting effects of fat and fructose may account for the outcomes observed under the HFFrD, in which fructose appeared to attenuate fat-induced weight gain, especially when consumed in solid forms.

In contrast to brain regions such as the hippocampus and hypothalamus, molecular alterations in the PFC induced by hypercaloric diets remain relatively understudied. Investigating PFC responses is particularly relevant given this region’s role in regulating feeding behavior [12–14], which is thought to contribute to the development of obesity. Although the underlying mechanisms are not fully understood, disruptions in neurotransmitter homeostasis—especially GABA signaling within the PFC—may influence the intake of energy-dense foods. Indeed, the ventromedial PFC has been shown to modulate impulsive eating through projections to the hypothalamus [64]. Previous studies in Osborne–Mendel rats also reported that fat consumption increases whole-brain GABA levels, potentially acting as a suppressive signal to limit the intake of highly palatable foods [65]. Recent evidence has shown both short- and long-term exposure to HFDs impair GABAergic signaling in the orbitofrontal cortex, a region implicated in food-related decision-making [66]. Similarly, studies have reported reduced cortical GABA levels in HFD-fed rodents and have proposed that these reductions affect feeding behavior and cognition [30, 31]. In this context, the consistent up-regulation of *Gad1* observed across all dietary groups in this study may reflect a potential engagement of GABAergic pathways within the PFC, with a possible role in the modulation of motivation toward hypercaloric food intake driven by palatability.

Among the distinct alterations associated with hypercaloric diets, those related to glucose metabolism may have important implications for brain dysfunction in diet-induced obesity, but precise measurement in vivo is still a challenge [23]. Nonetheless, evidence in HFD-fed rodents has previously shown an increase in transcript levels of *Slc2a1* [54] and a decrease in protein expression of GLUT1 transporters in the hippocampus [25], as well as in endothelial cells, which compromised brain glucose uptake [26]. Unfortunately, this type of evidence in the PFC is scarce, as it is research focused on HFrDs and HFFrDs. Some of these reports have found a downregulation of GLUT1 in the frontal cortex of rats exposed to HFFrD for 12 weeks [27, 28]. Based on our results, higher *Slc2a1* expression appears to be another consistent hallmark of excessive fat and fructose intake. Further molecular or neuroimaging analysis will help to determine if this change reflects a metabolic adaptation to optimize glucose metabolism in the PFC.

An important finding of this study is the differential regulation of *G6pd*, *Gck*, *Pck1*, and *Pcx*. These novel observations suggest these genes are diet-specific modulated, however, more research is needed to determine the role of these enzymes on the distinct adaptive responses and possible optimization of glucose metabolic pathways in the PFC, since such responses could be engaged by HFD and HFrD but attenuated under HFFrD. In the brain, glucose is metabolized by three main routes: glycolysis, the PPP, or the glycogen pathway [67], with gluconeogenesis also occurring in astrocytes [39]. However, the PPP and gluconeogenic pathways remain poorly characterized in models of diet-induced obesity. In the present study, we have not found significant changes in *G6pd* expression. Although the precise role of *Gck* in the PFC remains unclear, this kinase may function as a glucose sensor, regulating glycolytic activity by promoting glucose-6-phosphate formation, as described in pancreatic β-cells [68]. Further studies focused on cerebral *Gck* functions would provide valuable information to confirm such a hypothesis. On the other hand, the upregulation of *Pck1* suggests that excessive intake of fats or fructose may exert certain influence on gluconeogenic activity. In astrocytes, gluconeogenesis depends on phosphoenolpyruvate generated by PCK1 from oxaloacetate produced in mitochondria by pyruvate carboxylase, encoded by *Pcx* [39]. Notably, *Pcx* expression was selectively increased by HFrD, identifying the overexpression of this gene as another signature underlying diet-specific effects on amino acid homeostasis within the glutamate–glutamine–GABA cycle.

Moreover, glucose serves as a fundamental substrate for generating precursors required for neurotransmitter and amino acid synthesis, processes essential for brain functions such as neuroplasticity, learning, and memory [69]. As neuronal activity increases, cerebral glucose demand rises accordingly, necessitating coordinated adjustments in cerebral blood flow, glucose transport, and metabolism to support neurotransmitter homeostasis [70]. As demonstrated in the present study, these metabolic adaptations are sensitive to excessive intake of saturated fats or fructose. Notably, the PFC appears more resistant to HFD-induced alterations than the hippocampus, which exhibits a distinct pattern of amino acid and gene expression changes under similar dietary conditions [54]. Nevertheless, HFFrD markedly disrupted glutamine and glutamate synthesis in the PFC, leading to reduced levels of these neuroactive amino acids, a finding further supported by concomitant decreases in alanine. In contrast, HFrD was associated with patterns suggestive of enhanced glycolytic activity, with pyruvate potentially redistributed toward interconnected metabolic pathways, including the glutamine–glutamate–GABA cycle, as well as others like the methionine-homocysteine shuttle. Consistent with this interpretation, expression of *Irs2*, a key regulator of cerebral energy balance and glucose metabolism [71], was increased under the HFrD.

HFD and HFrD are widely known to impair insulin sensitivity [72–75]. Some research has even found that excessive fat intake on a single day can be severely detrimental to whole-body insulin sensitivity in healthy young adults [71] and that moderate consumption of fructose or sucrose impairs hepatic insulin sensitivity in young male adults [76]. Moreover, severe impairments in insulin response have been reported after 8 weeks of a combined HFD and high-sucrose diet in young adult male mice [77]. A recent study showed that the relationship between peripheral insulin resistance and brain glucose metabolism depends on subjects’ metabolic conditions [78]. For instance, in individuals with severe obesity and impaired glucose tolerance, insulin stimulation increases brain glucose metabolism, but not in the fasting state [79]. In this context, consumption of hypercaloric diets may exert sustained changes in brain glucose metabolism that are modulated by peripheral insulin signaling. Importantly, it could vary with type of diet, thereby explaining the differential effects observed in this study. For this reason, further examination of insulin resistance and glucose tolerance under the conditions of this study will provide valuable complementary information to better understand diet-specific changes in glucose and amino acid metabolism.

Substantial evidence indicates that hypercaloric diets adversely affect cognitive functions, including memory [10, 19, 80]. However, the individual contributions of saturated fats and sugars, particularly fructose, remain incompletely understood. In a previous study, we showed that 10 weeks of HFFrD, but not HFD alone, impaired recognition memory in mice, accompanied by neurochemical changes in the hippocampus. Medial PFC connections with the hippocampus have been shown to modulate novelty discrimination [60], suggesting that neurochemical alterations in the PFC may contribute to the modulatory activity of recognition memory by the hippocampus. However, that work did not include an HFrD group, limiting the interpretation of fructose-specific effects. Although fructose has been implicated in cognitive dysfunction, including recognition memory deficits [80], the present findings indicate that HFrD alone did not impair recognition memory in adult male mice. Ross et al. [81] reported spatial memory deficits only after prolonged exposure (18 weeks) to diets with substantially higher fructose content than used here. Importantly, our results do not suggest that HFrD is benign, as fructose intake induced hyperglycemia independently of adiposity. These findings support the notion that metabolic disturbances and cognitive impairments induced by hypercaloric diets may emerge in a time-dependent manner.

Previous findings have consistently reported that high consumption of fat and sugar decreased locomotor activity in the open field [77]. Consistently, we observed this effect in our mice, which was related to the specific consumption of combined HFFrD. In particular, fructose has been shown to reduce locomotor activity in rodents without affecting cognition [82]. A possible explanation for this is that prolonged sugar intake leads to sarcopenia through alterations such as impaired insulin sensitivity [83]. We hypothesized that impaired motor-cognitive behavior in mice exposed to diets high in fat and sugar may be related to the detrimental effects of fructose on muscle tissue and to synergistic noxious effects of fats and fructose on mechanisms involved in memory processing. However, further studies are needed to determine the effects of such factors on performance in cognitive tasks requiring exploratory activity.

The synthesis and regulation of neuroactive amino acids such as GABA, glutamine, and glutamate during memory-related processes are critical for optimal cognitive performance. For example, an increased glutamate/glutamine ratio in the medial PFC has been shown to enhance functional connectivity among the PFC, hippocampus, and thalamus, and this connectivity correlates with improved episodic memory performance [84]. Disruptions in the glutamate/GABA–glutamine cycle have also been reported in animal models of Alzheimer’s disease [85], potentially contributing to the pathophysiological mechanisms underlying memory impairment. In addition, GABAergic and glutamatergic signaling within the PFC plays a modulatory role in hippocampal-dependent memory formation [59]. Taken together, these findings suggest that alterations in neuroactive amino acid homeostasis in the PFC induced by hypercaloric diets may compound detrimental effects on neurotransmission, thereby compromising episodic-like memory processing.

The present study has several important limitations. First, HPLC measurements reflect global amino acid concentrations in PFC homogenates, precluding determination of whether the observed changes arise from specific cell types (e.g., astrocytes vs. neurons), subcellular compartments (cytosol vs. organelles), or discrete PFC subregions (e.g., medial or infralimbic PFC). Second, a mechanistic interpretation of amino acid alterations would benefit from additional methodological approaches, such as protein quantification, since mRNA transcript abundance correlates only partially with protein abundance, typically explaining about one to two-thirds of the variance in protein levels [86]. Moreover, direct tracing of glucose carbon flux, particularly because several of these metabolites participate in multiple interconnected metabolic pathways. This could be addressed in future work by using ^13^C-labeled glucose combined with magnetic resonance spectroscopy to quantify pathway-specific metabolic contributions. Nonetheless, our findings are broadly consistent with prior studies employing similar approaches. For example, fructose intake has been reported to increase astrocytic TCA cycle activity and elevate whole-brain glutamate, glutamine, and GABA [87]. Conversely, a 60% kcal HFD has been shown to increase brain glucose levels without altering cortical concentrations of glutamate, glutamine, GABA, aspartate, glycine, alanine, or taurine in mice [88]. Another limitation of this study is the exclusive use of male animals, which limits the generalizability of the findings. Given known sex-specific differences in neurochemistry and metabolism, the absence of females prevents assessment of sex-dependent effects. Therefore, results should be interpreted within the context of male physiology. Future studies should prioritize inclusion of both sexes to provide a more comprehensive understanding and facilitate effective translation of findings to diverse populations.

Some of our results did not align with previous studies which may involve several factors. It is noteworthy that the diet composition, time duration, and brain area analyzed may explain the variability observed in the literature on diet-induced obesity models. Moreover, other factors—such as age, sex, species, rodent strain, and, importantly, the form in which foods are administered (solid or liquid), as well as the stability of diet composition—may also contribute. Thus, the high variability among studies on the impact of hypercaloric food on molecular changes (such as amino acid levels and gene expression) related to cognitive dysfunctions like memory likely results from the multiple influences of diet-induced obesity in rodents. The present findings should therefore be interpreted in the context of the conditions established in this study.

In conclusion, this study demonstrates that saturated fat and fructose exert distinct—and sometimes opposing—effects on systemic metabolism and PFC molecular homeostasis. These results provide evidence that the composition of hypercaloric diets critically shapes PFC metabolic adaptations and neurotransmitter-related amino acid homeostasis, with potential implications for diet-associated cognitive decline.

## Data Availability

The data that support the findings of this study are available upon request.
